# Experimental evidence for yawn contagion in orangutans (*Pongo pygmaeus*)

**DOI:** 10.1038/s41598-020-79160-x

**Published:** 2020-12-17

**Authors:** Evy van Berlo, Alejandra P. Díaz-Loyo, Oscar E. Juárez-Mora, Mariska E. Kret, Jorg J. M. Massen

**Affiliations:** 1 Institute of Psychology, Cognitive Psychology Unit, Leiden University, Leiden, The Netherlands; 2 Leiden Institute for Brain and Cognition (LIBC), Leiden, The Netherlands; 3 Laboratorio de Ecología de La Conducta, Instituto de Fisiología, Benemérita Universidad Autónoma de Puebla, Puebla, Mexico; 4 Department of Biology, Animal Ecology Group, Utrecht University, Utrecht, The Netherlands

**Keywords:** Social evolution, Psychology and behaviour

## Abstract

Yawning is highly contagious, yet both its proximate mechanism(s) and its ultimate causation remain poorly understood. Scholars have suggested a link between contagious yawning (CY) and sociality due to its appearance in mostly social species. Nevertheless, as findings are inconsistent, CY’s function and evolution remains heavily debated. One way to understand the evolution of CY is by studying it in hominids. Although CY has been found in chimpanzees and bonobos, but is absent in gorillas, data on orangutans are missing despite them being the least social hominid. Orangutans are thus interesting for understanding CY’s phylogeny. Here, we experimentally tested whether orangutans yawn contagiously in response to videos of conspecifics yawning. Furthermore, we investigated whether CY was affected by familiarity with the yawning individual (i.e. a familiar or unfamiliar conspecific and a 3D orangutan avatar). In 700 trials across 8 individuals, we found that orangutans are more likely to yawn in response to yawn videos compared to control videos of conspecifics, but not to yawn videos of the avatar. Interestingly, CY occurred regardless of whether a conspecific was familiar or unfamiliar. We conclude that CY was likely already present in the last common ancestor of humans and great apes, though more converging evidence is needed.

## Introduction

Yawning is an evolutionarily old phenomenon as its associated motor features can be recognized in different groups of animals^1^. It follows a stereotyped pattern that, once started, is unstoppable^2^. Apart from its spontaneous form, it is also notoriously contagious, at least for some species; i.e. individuals yawn as an unconscious and automatic response to seeing or hearing other individuals yawn^3^. While a yawning-like pattern is observed in a wide range of vertebrates^1^, contagious yawning (CY) is less wide-spread. To date, CY appears to be present in only a few, generally social species, including tonkean macaques^4^ (and possibly stumptail macaques^5^), gelada baboons^6^, chimpanzees^7–14^, bonobos^15,16^, dogs and wolves^17–19^, sheep^20^, elephant seals^21^, budgerigars^22^, and rats^23^. In contrast, studies failed to show CY in grey-cheeked mangabeys and long-tailed macaques^24^, mandrills^1^, common marmosets^25^, lemurs^26^, horses^27^, lions^1^, tortoises^28^, and fish^1^, even though some of these species are also very social. Despite growing interest in CY, both its proximate mechanisms (how it functions and develops) and ultimate causes (why and how it evolved) currently remain unclear.

Several hypotheses have been put forward, following a Tinbergian approach^29^. One view on the proximate mechanism underlying CY is that it is an automatic form of physiological or emotional state-matching between individuals. This synchrony of states between individuals may work via a perception–action mechanism (PAM), an adaptive mechanism that serves to create and maintain relationships in highly social species and that can give rise to higher-order cognitive phenomena such as empathy^30^. Some scholars argue that CY taps into the same PAM as emotion contagion (e.g.^6,7,31,32^), which is the tendency to automatically synchronize emotional states with another individual^33^. Following this line of thought, CY can thus potentially be a proxy for empathy (i.e. the CY-empathy hypothesis)^6,9,12,18,31,34,35^. Indeed, neuroimaging studies have shown increased brain activity during CY in areas involved in theory of mind and social cognition ^36–38^, corroborating the idea that CY is linked with emotional state-matching and perhaps even empathy. Furthermore, individuals who score low on empathy scales (e.g. individuals on the autism spectrum) are less likely to engage in CY^39^, and females yawn more frequently in response to seeing others yawn than males do, reflecting the idea that females show higher levels of empathy than males because of their investment in offspring care^40^. Nevertheless, there are some studies that do not find such a clear link between CY and empathy. For instance, when people with autism spectrum disorder (ASD) are instructed to pay attention to the eyes (avoidance of the eyes is one of the characteristics of ASD), they are just as likely to yawn contagiously as neurotypical individuals^41^. Furthermore, the gender bias is not consistently found (e.g. ^10,42^) and heavily debated^43,44^. For instance, in chimpanzees, it appears that males yawn more frequently than females in response to seeing other males yawn^10^. Finally, while dogs do engage in CY, its presence is not affected by whether the yawner is prosocial versus the yawner being antisocial^45^. The mixed findings in the studies investigating the relationship between CY and a complex construct such as empathy show that the topic deserves more attention, and that it is still debated (see Massen & Gallup (2017) for a critical review).

The emotional bias hypothesis is a more detailed specification of how CY can be socially modulated through a shared PAM, namely via social closeness and familiarity. The hypothesis predicts that individuals who are socially, and thus emotionally close are also more likely to yawn contagiously in response to each other^15,16,18,19,34,46,47^. Additionally, individuals from a group (i.e. familiar others) are more likely to yawn in response to each other than to unfamiliar others^9,18^. A potential issue that has been raised is that these studies often fail to rule out simple alternative explanations for CY that do not require higher-order cognition^48^. For instance, effects of familiarity on CY may be explained by a general tendency to bias attention to familiar and socially close others^48^. Nevertheless, in a recent study investigating auditory yawn contagion in humans, yawns were most contagious between family and friends while controlling for the potential effects of increased attention to socially close others using a non-visual stimuli^34^. Still, in quite some social species, the linkage between CY and social closeness or familiarity is not found^10,45,49–51^. For example, a recent study analyzing a large dataset on CY in dogs shows CY is present in dogs, but is not affected by familiarity or other potential mediators such as sex or prosociality^45^. It therefore remains possible that mechanisms other than the same PAM that underlies emotion contagion or empathy are mediating CY. For instance, CY may result from stress induced by a common stressor in the environment^5,52^. Thus, rather than being mediated by seeing others yawn, yawning occurs as a response to the stressor. Individuals that are stressed are known to show higher rates of self-directed behaviors, of which yawning and scratching are examples^53^, and indeed, in one study involving stumptail macaques, monkeys yawned more frequently in response to a video clip of yawns as compared to a control, but also scratched more^5^. The authors concluded that tension was most likely mediating the occurrence of yawning in the yawn condition. In short, while it is likely that CY is a social phenomenon, its exact mechanisms remain an active field of investigation.

Notwithstanding the debate on proximate mechanisms, little attention has been given to more ultimate explanations for CY. One of the few hypotheses out there is that CY is an adaptive mechanism that helps with social coordination^54^. Accumulating evidence suggests that yawning itself serves to cool the brain as to maintain homeostasis^55–60^ and consequently may increase alertness and aid in vigilance. Within this social coordination hypothesis, CY, in turn, may help to spread vigilance within the group, for instance to remain alert for potential predators^54,57^. Specifically, it may be adaptive to match the state of a vigilant conspecific as it may have sensed a predator, which the individual itself did not yet sense. To date, however, the social coordination hypothesis remains untested, and the thermoregulatory function of yawning is still debated (e.g.^61,62^, but see^58^ for a response to the critique).

Another fruitful way to explore evolutionary hypotheses is through phylogenetic comparisons. Palagi et al. (2019) proposed the *common trait among hominids* hypothesis which states that, given the shared phylogeny between humans and great apes, CY may find its roots in a shared underlying socio-cognitive mechanism that was already present in at least the last common ancestor (LCA) of all hominids. Moreover, since CY is also present in some Old-World monkeys and non-primate species, its roots could be much older, or CY is an example of convergent evolution. To date, few data exist to perform comparisons and most interestingly, the picture among the great apes is not yet clear. There is convincing evidence for CY in chimpanzees^7,8,10,12,14^. In bonobos, two observational studies^15,16^ and an experiment^63^ show clear evidence for CY, while one experimental study did not^12^. However, the latter study only tested four individuals, thus making it very likely that CY is, indeed, present in bonobos. Finally, the first comprehensive study on gorillas combining an experimental and naturalistic approach found no evidence for CY^64^. Notoriously absent are data on CY in orangutans, which, considering their semi-solitary lifestyle^65^ may be of comparative interest for a social phenomenon like CY. To date, the only existing study involving orangutans failed to find evidence for CY^12^, yet the sample size was too small to be conclusive. In general, orangutans in the wild roam mostly solitarily: males travel alone, and mothers travel with their offspring^66^. Due to overlapping home ranges, occasional encounters and affiliation are possible, but generally do not occur frequently^66,67^. Consequently, finding out whether CY is present in orangutans will further help elucidate the hypotheses previously discussed.

The current study attempts to clear up the picture of CY in hominids in two ways. First, we aim to find a convincing answer to whether CY is present in orangutans or not via an experimental design involving the presentation of yawning and neutral stimuli of orangutans to 8 orangutans. Second, we also investigate whether this potential yawn contagion is affected by a familiarity bias, i.e. whether CY is stronger between individuals that know each other versus unfamiliar individuals. To this end, we exposed orangutans to videos showing either yawn or control clips of familiar (i.e. conspecifics living in close proximity) and unfamiliar orangutans, as well as a 3D avatar^68^ and measured their response (yawns). Additionally, we also measured the occurrence of scratching to rule out potential effects of stress on the occurrence of yawning^53^. So far, CY appears to be exclusively present in highly social species, and because orangutans do not show high affiliative tendencies, we therefore expected that orangutans do not show CY.

## Materials and methods

### Ethics

The care and housing of the orangutans was adherent to the guidelines of the EAZA Ex situ Program (EEP). As the study was non-invasive in nature, there was no need for the approval of the Ethics Committee of Apenheul primate park and the study complied with the requirements of the Dutch Animal Care and Use Committee.

### Subjects

Eight orangutans (ages: 15 months-36 years, 4 males) housed at Apenheul (The Netherlands), were tested (see Table 1 for an overview on sex and age). Individuals were divided over four neighboring enclosures and group composition varied weekly (Figure S1). The two adult females that had dependent offspring were always housed with their offspring and sometimes with one adult male. Experiments took place in the visitor area but while the park was closed to visitors. We tested individuals using a movable 47″ TV (LG 47LH5000, 1920 × 1080 pixels) placed in front of the enclosures to which the orangutans were habituated before commencing testing. The screen was always directed at one of the four enclosures, which prevented orangutans in the other enclosures from seeing the videos. Food was provided four to six times a day and consisted of a variety of vegetables, and sometimes nuts, hay, and fruit, hidden in the enclosure for foraging purposes. Water was available ad libitum.

**Table 1:** Overview of test subjects, their sex, age, and relationship.

Name	Sex	Date of Birth	Developmental Stage^1^	Relationship
Amos	Male	20-12-2000	Adult	Father of Kawan, Baju and Indah
Baju	Male	02-12-2015	Juvenile	Son of Amos and Wattana
Indah	Female	19-10-2017	Infant	Daughter of Amos and Samboja, granddaughter of Sandy
Kawan	Male	22-02-2010	Adolescent (unflanged)	Son of Amos and Wattana
Kevin	Male	~ 1982	Adult	Born in the wild, no kin in group
Sandy	Female	29-04-1982	Adult	Mother of Samboja, grandmother of Indah
Samboja	Female	09-01-2005	Adult	Daughter of Sandy, mother of Indah
Wattana	Female	17-11-1995	Adult	Mother of Kawan and Baju

^1^At start of experiment (January 2019).

### Stimuli

**Figure 1:**
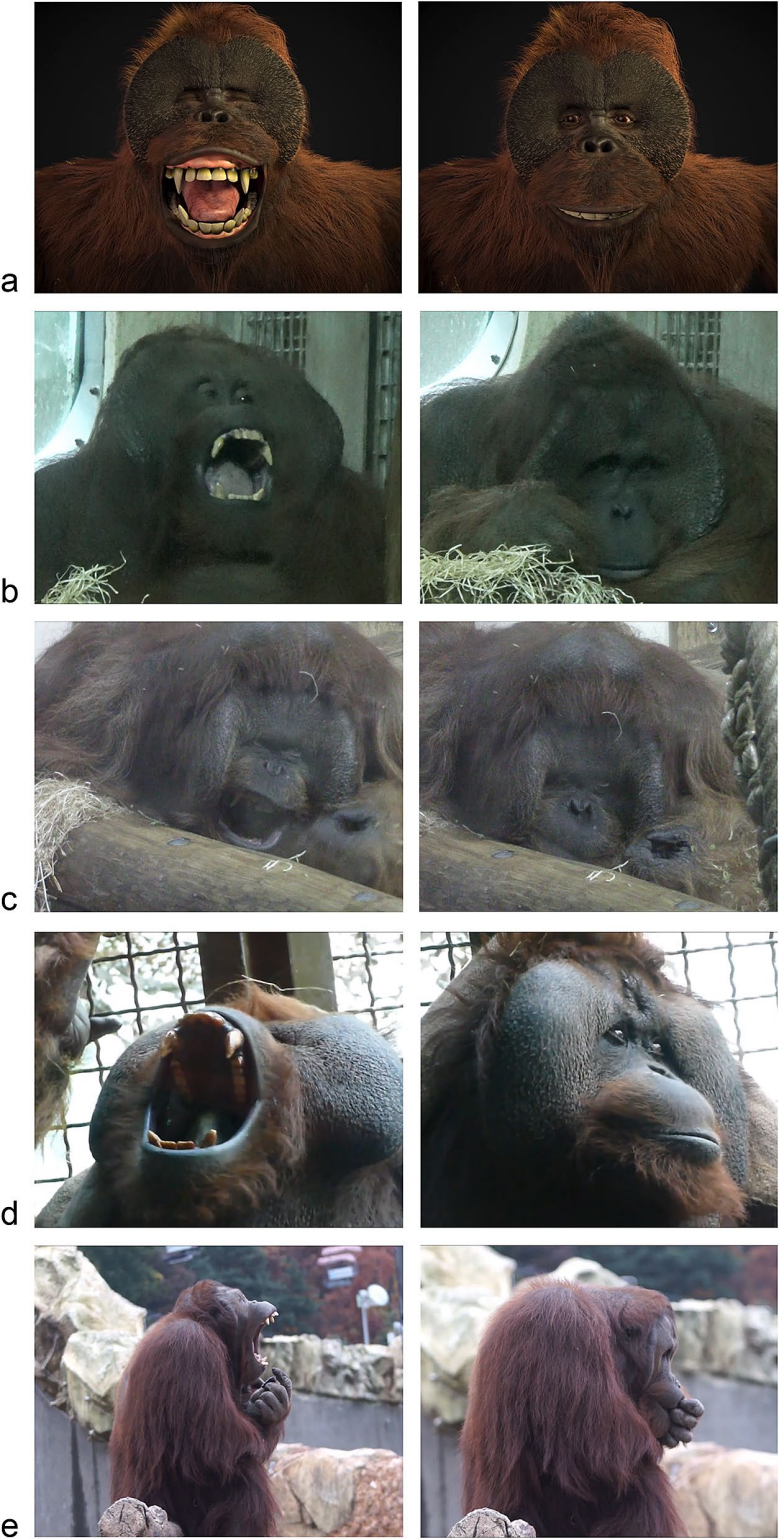
Stills of the videos used in the experiment with yawns on the left and controls on the right. A: Avatar*^68^, B: Familiar adult 1, C: Familiar adult 2, D: Unfamiliar adult 1^70^, E: Unfamiliar adult 2^71^. *To decrease the chances of pseudo-replication within this category we created horizontally mirrored copies of the yawn and control videos of the avatar such that – similar to the other triggers – we had two yawn and two control videos in total.

The experiment involved three categories of mute, full-screen videos, each consisting of both a yawn and control condition (see Fig. 1 for examples). We used mute videos as the enclosures were sealed with thick glass that dampened most of the sound both ways. Yawn videos showed clear yawns either filmed from the front or side, whereas control videos consisted of individuals with a neutral face and in a relaxed body position. Both types of videos involved movement, with yawn videos showing a wide gaping of the mouth followed by a relaxation of the mouth and jaw^69^, including display of the teeth, and control videos showing an individual with a closed mouth with random movements of the lips. Both control and yawn videos were always of the same individual, and therefore the body position and face were identical. The *familiar* video category consisted of two adult males housed in the zoo (published under CC BY-NC-SA). For the *unfamiliar* video category, we used two adult males taken from clips on YouTube^70,71^(published under the YouTube Standard License). Finally, in the *avatar* video category we used two mirrored videos of a computer-generated adult male. The 3D orangutan was created by Paul Kolbrink^68^ from XYZ-Animation and designed in Autodesk 3ds Max (2017) using the Octane render engine (published under CC BY-NC-SA). Using these videos, we created video sequences starting with a primer video that depicted caretakers beckoning the orangutans towards the TV screen, which were created to grab the orangutans’ attention right before the start of a trial. As we repeated the presentation of our video database four times during the course of the experiment, there were four different primers; one for every repetition.

### Procedure

The experiment was carried out between 21–01-2019 and 13–03-2019. In this period, the park was closed for visitors. A test session involved the presentation of two different trials, each consisting of a specific video sequence, and each trial followed by an observation period. The video sequence consisted of a primer, followed by either a yawn or control video (lasting 14 s), which was repeated 4 times and with a colored screen (again to grab attention) for 1 s in between each video. The length of one video sequence was thus 90 s (cf. Massen et al., 2013): primer (30 s) – colored screen (1 s) – yawn/control video (14 s) – colored screen (1 s) – yawn/control video (14 s) – colored screen (1 s) – yawn/control video (14 s) – colored screen (1 s) – yawn/control video (14 s). The presentation of one video sequence (representing one trial) was then followed by a 3.5-min observation period, after which the second trial started. If the first trial involved yawn videos, the second trial involved control videos and vice versa. The second trial was also followed by a 3.5-min observation period, completing one test session. Within one test session we always showed the same stimulus individual.

We cycled through the entire video database four times (i.e. 4 blocks) over the course of the experiment to ensure sufficient data points. The order of control and yawn trials were counterbalanced per subject, and was further counterbalanced over the subjects per block. Within each block, *trigger* (i.e. familiar/unfamiliar/avatar) was also randomized per subject. We designed a testing schedule based on eight test subjects, but two of those subjects involved a mother-infant pair and a mother-juvenile pair in which the infant/juvenile never left the mother. As such, we created a test schedule for six individuals rather than eight. With these six test subjects, three types of triggers, two conditions (yawn and control), two orders of condition presentation (yawn-control, or control-yawn), and finally four repetitions, we had a total of 288 test sessions and 576 trials planned (see Table S1a-d and S2 for an overview). However, one video sequence was accidentally presented an extra time, resulting in 289 rather than the planned 288 sessions after data collection finished. On any given testing day, individuals participated in one or two sessions with 30 min breaks between video presentations to the tested subject. Furthermore, subjects never saw a video sequence more than once on any given day.

APDL and OEJM recorded all occurrences of yawning and scratching, and scratching was recorded as a measure for arousal and tension^53^. It was not possible to reliably quantify the amount of time spent looking at the screen due to the lack of continuous visibility of the gaze of the orangutans. To nonetheless ensure maximum attention to the screen, we presented primers before video sequences and colored videos in-between yawn and control clips, and we only started testing when orangutans had a direct line of sight towards the screen. Additionally, before each trial, we observed the orangutans for five minutes, and only started a trial if there were no yawns before the presentation so as to rule out that yawns within a trial were potentially caused by a previous yawn outside of the trial. Furthermore, yawns were scored in response to either the yawn or control video only if a subject looked at least once to the screen during presentation. If bystanders in the same enclosure attended to the screen, their behaviors were also scored. Data collection ended after 10 min, concluding one test session. Finally, EvB coded 15% of the videotapes for inter-rater reliability purposes. Results showed a good agreement on occurrences of yawning (ICC = 0.764, *p* < 0.001 Table S3) and scratching (ICC = 0.894, *p* < 0.001, Table S4). In subsequent analyses, only yawns on which the raters agreed were used.

### Statistical analysis

The dependent variable was whether a subject yawned in response to a video or not. Because it is difficult to disentangle between whether multiple yawns occurring in succession are caused by another individual, or whether they are simply the result of an urge to yawn multiple times perhaps because of self-contagion (i.e. where your own yawns cause you to yawn again), we did not compare rates of yawning to establish CY^72^. Rather, we looked at the likelihood of yawning within the yawn and control condition to establish the presence or absence of CY in orangutans. Nevertheless, when contagion indeed occurred, yawning rate could inform about the *strength* of contagion^72^. As such, we analyzed our data using hurdle models in R (lme4 package). Hurdle models follow a two-step method that first deals with zero-inflated count data and subsequently with positive counts once the initial hurdle is crossed^73^, which make them applicable to our dataset.

In the first hurdle model we focused on whether CY is present or absent in orangutans by comparing the likelihood of yawning in the yawn and control condition using a binomial GLMM, in which we added *condition* as a fixed effect and *subject* nested in *trial* as a random effect. In the second step of the model, we analyzed the rates of yawning using a negative binomial GLMM only in those cases where at least one yawn occurred. Again, we entered *condition* as a fixed effect and *subject* nested in *trial* as a random effect. In the second hurdle model, we tested for potential effects of both *condition* and *trigger* (i.e. familiar/unfamiliar/avatar) and their interaction on the likelihood of yawning using a binomial GLMM, entering *condition* and *trigger* and their interaction as fixed effects, and again *subject* nested in *trial* as random effect. In the second step of the model, we were interested in how the conditions and triggers affected yawning rates in those cases that at least one yawn occurred. To investigate this, we entered *condition* and *trigger* and their interaction as fixed effects and *subject* nested in *trial* as random effect using a negative binomial GLMM.

It is possible that the likelihood of yawning in the conditions is due to the stimuli somehow being arousing to the observers, complicating the interpretation of the underpinnings of CY (see e.g.^5^). For instance, yawning often involves display of the canines, which may be arousing for the orangutans^74^. Therefore, as a control analysis, we looked at self-scratching behavior as this is indicative of arousal in primates^53^. In a third hurdle model, we checked whether the likelihood of scratching is affected by *condition* (fixed factor), with *subject* nested in *trial* as random factor and using a binomial GLMM. In the second step of the model using a negative binomial GLMM with *subject* nested in *trial* as random factor, we investigated whether scratching rate was affected by *condition, trigger*, and their interaction as fixed factors only in those cases when scratching occurred.

In all analyses, we compared the models to their respective null-models (i.e. including only the random effects) and only report on significant values if the models and null-models differ significantly from each other^75^. For post-hoc contrasts of interaction effects we report corrected p-values using Tukey-adjustments. Alpha was set to 0.05.

## Results

In total, we witnessed 83 yawns across 8 individuals and 289 sessions. First, we investigated the likelihood of yawning in the two conditions. We found a significant effect of *condition*; yawning was more likely to occur in the yawn versus the control condition (*β* = 3.45, *SE* = 1.06, *p* = 0.001). Next, we compared the yawning rate between the two conditions in those cases that at least one yawn occurred, but this alternative model did not deviate significantly (*χ2*(1) = 3.09, *p* = 0.079) from its respective null-model.

**Figure 2:**
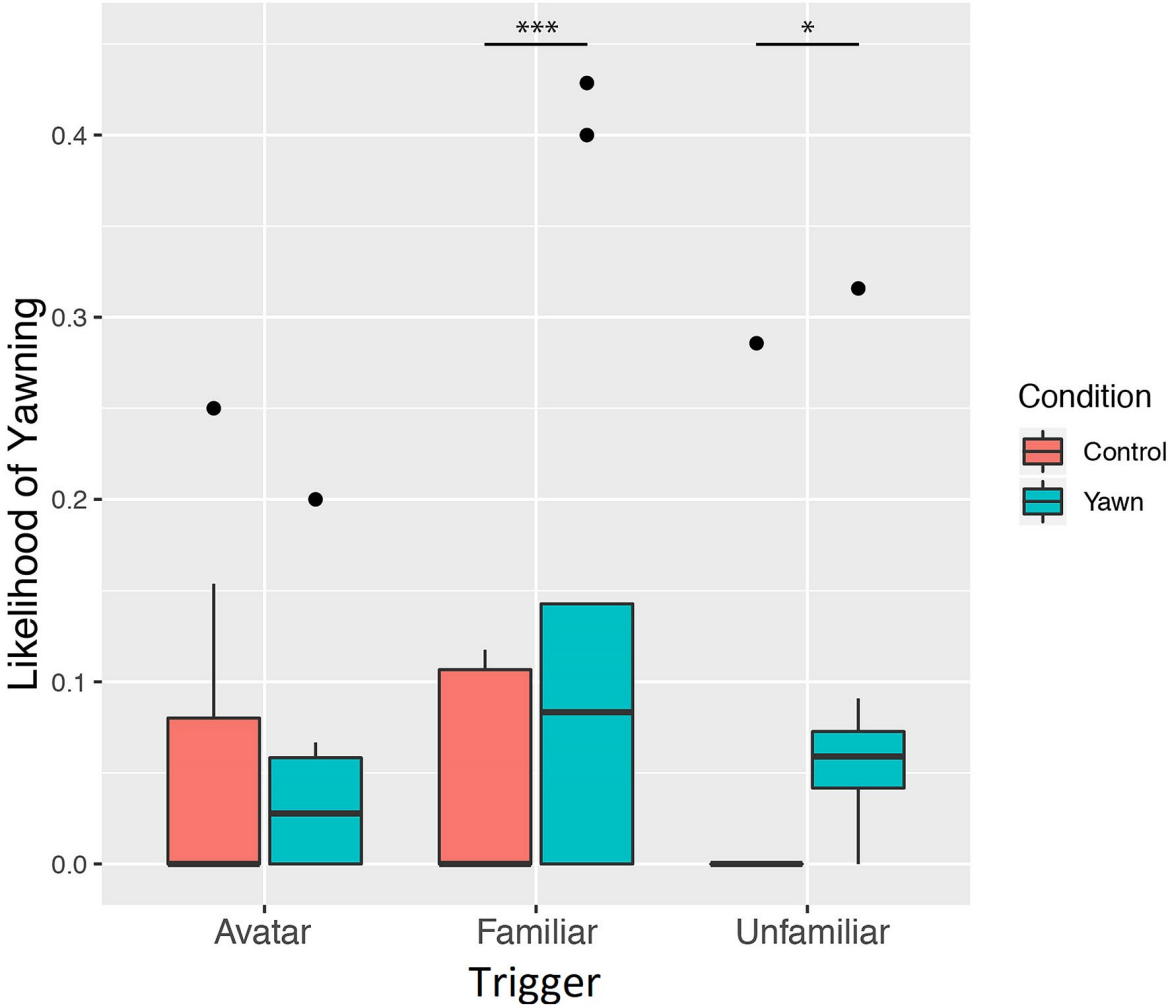
Likelihood of yawning across conditions and triggers. Boxplots show the median (solid line), 25th-75th percentile (box) and the largest and smallest value within 1.5 times the interquartile ranges respectively (whiskers). Dots reflect outliers.

Assessing whether familiarity affects the occurrence of CY, we found a significant interaction effect of *trigger* (familiar, unfamiliar, avatar) with *condition*. Specifically, we found a significant contrast of yawns between the yawn and control condition in the familiar (*β* = 6.62, *SE* = 1.59, *p* < 0.001) and unfamiliar trigger (*β* = 3.45, *SE* = 1.52, *p* = 0.023), but not in the avatar trigger (*β* = 0.09, *SE* = 1.58, *p* = 0.950) (Fig. 2). Hence, orangutans are more likely to yawn in response to yawning videos rather than to control videos, but only when the yawning individual is a ‘real’ orangutan (i.e. a familiar or unfamiliar conspecific), and are less likely to yawn in response to the avatar. To investigate whether the likelihood of CY differed with regard to familiarity with the ‘real’ orangutan stimuli, we also ran an additional binomial model on a reduced dataset that excluded all trials with the avatar (see supplemental materials). Whereas this model confirmed the previously found effect of *condition*, here we did not find a significant interaction between *condition* and *familiarity*, suggesting that the likelihood of CY was not being modulated by the familiarity with the ‘real’ orangutan. We also investigated the effect of *familiarity* on yawning rate using the same reduced dataset, but the model including the interaction between *condition* and *trigger* did not significantly improve the null model (*χ2*(3) = 3.50, *p* = 0.321). As such, while we can establish that orangutans do show CY in response to yawn videos of familiar and unfamiliar conspecifics, this likelihood of CY is not modulated by familiarity and we cannot draw any conclusions regarding the *strength* of CY in relation to familiarity.

Looking at scratching, we first investigated the likelihood of scratching when viewing yawning and control videos, and found that the *occurrence* of scratching did not differ between conditions (*β* = -0.17, *SE* = 0.17, *p* = 0.319). Similarly, scratching *rates* were not significantly higher in the yawn versus control condition (*β* = 0.10, *SE* = 0.09, *p* = 0.301). Moreover, both models did not deviate significantly from their null-model (*χ2*(1) = 1.06, *p* = 0.303). Hence, it is unlikely that orangutans were more aroused viewing yawn videos compared to viewing control videos, at least when measured via scratching Additionally, we also included scratching in our original models on yawning as a covariate, and found it to not significantly explain the likelihood of yawning, nor to influence the found effects of condition and the lack thereof in the avatar treatment (see Supplements).

## Discussion

Here we find that orangutans yawn contagiously in response to conspecifics yawning, independent of whether the conspecific is a familiar or unfamiliar individual. Furthermore, orangutans were not susceptible to yawns of an avatar. Additionally, the videos used in our experiment appeared to be similarly arousing. That is, there was no difference in scratching (an indicator of stress) between the conditions. We here discuss the consequences of our findings for the different proximate and ultimate hypotheses that currently exist.

CY has thus far been observed in highly social species^6,7,15,17,19–22^ (but see:^1,24–27^). Orangutans have meaningful social interactions that occur more often than is expected by chance alone^76^, but these interactions occur at a much lower frequency compared to bonobos and chimpanzees^66,67^. Interestingly, our results show that orangutans exhibit CY, suggesting that a high degree of affiliation within a species is not necessary for CY to occur. This also indicates that more studies are needed that investigate the presence or, importantly, absence of CY in a variety of species that differ on their social organization and affiliative tendencies. At the same time, it has to be noted that our sample consists of zoo-housed orangutans that were also born in captivity. In captivity, frequencies of affiliation can exceed those observed in the wild^77^, thus potentially increasing the likelihood of CY to occur. Nevertheless, our results do show the presence of CY in orangutans and the few generations of zoo-living individuals cannot inform us about any selection pressures that have resulted in this tendency in orangutans. Our results must therefore be discussed in light of the orangutans’ natural behavior and social environment.

In our study, we did not find an effect of familiarity on CY, suggesting that at least in orangutans, social modulation of CY may not be present. While presence of social modulation of CY is often used as confirmation of CY and emotion contagion sharing the same underlying perception–action mechanism^9,15,16,18,35^, its absence in our data makes it more difficult to interpret the emotional bias hypothesis. Orangutans do have some preferences when it comes to their interaction partners, thus one could expect social modulation of CY under the emotional bias hypothesis. For instance, related female orangutans are known to associate more often than unrelated females^78^, and prefer the long-calls of dominant males^79^. Additionally, in a recent study, orangutans were shown to scratch contagiously in response to conspecifics scratching, suggesting a potential case of emotion contagion^80^. Interestingly, scratch contagion was stronger between weakly bonded individuals during tense situations, which shows a social closeness bias in the opposite direction. This suggests that a familiarity bias may be more flexible depending on the situation individuals are in (e.g. relaxed versus stressful contexts) and the nature of the behavior that is copied (e.g. scratching as an expression of tension). At the same time, there are other studies on highly social species that do not show a familiarity bias (e.g. chimpanzees^10^, dogs^45^, macaques^24^, and marmosets^25^). As such, there may be (currently unknown) species-specific traits that determine whether a familiarity bias occurs or not. The exact (social) function of CY remains unclear and thus alternative explanations that do not involve the PAM that is underlying empathy may still be possible (e.g. spreading of vigilance). As has been pointed out by others, solving this issue requires a more systematic study of CY that includes a bigger variety of animals, including solitary animals such as reptiles and amphibians^48^.

From an evolutionary perspective, our results pose an interesting conundrum: while we found CY in orangutans, it is not present in gorillas, even though the split between orangutans and other hominids is evolutionarily older than the split between gorillas and other hominids^81^. It is possible that the number of trials in the study by Palagi et al. (2019) were not sufficient to detect CY, as in our study, even with a large number of trials, we only detected yawns in 11.9% of all cases. Nevertheless, studies with chimpanzees that have few trials were able to establish CY in the past, albeit with a relatively large number of subjects^8,10,12^, and there was also no evidence for CY in naturalistic observations in gorillas^64^. Interestingly, it has been argued that in the past, orangutans may have been more social, but that due to long periods of low food availability, orangutan gregariousness was no longer viable^82^. This may suggest that the ancestor of all apes already possessed the mechanism underlying CY. However, based on observational and relatedness data, it has been suggested that this hominid lived in a group with gorilla-like structure in which one male could monopolize multiple females^82^. In this sense, it is difficult to explain why, given a similar social structure, gorillas do not show CY and orangutans do. It is possible that CY was somehow lost in the gorilla lineage, or that CY developed multiple times over the course of evolution. The loss of CY is theoretically possible, given that CY has been found in some, but not all primates^1,64^. Here, there is a role for the type of social system that characterizes a species in the loss (or occurrence) of CY^64^. There is, however, not yet enough variation in data on CY in different species of primates to draw clear conclusions. Furthermore, it is possible that the measures to detect CY in certain species are simply not sensitive enough. All these explanations can be true, given that the occurrence of CY is highly variable in primates in general. It is clear that more studies are needed in order to draw robust conclusions about the evolution of CY.

In our study orangutans did not significantly respond to the avatar, which contrasts with findings in chimpanzees^8^. Potentially, orangutans experienced the uncanny valley phenomenon in which the avatar looks very realistic, yet fails to behave like a real orangutan, therefore violating natural expectations of orangutan behavior. Indeed, previous research on monkeys showed that they preferentially looked at real or completely unrealistic 3D model monkeys compared to very realistic 3D models^83^. Nevertheless, this would likely have increased scratching when viewing the avatar, which was not evident in our study. Furthermore, a recent study investigating the uncanny effect in macaques showed that looking times did not differ between the Primatar (3D monkey head) and real or unrealistic images, indicating that the use of virtual stimuli can still be a promising way to study social cognition^84^. Future studies will have to verify whether the lack of evidence for CY using an avatar in our study is because the effect is truly absent, for instance by looking specifically at how similarity with another individual (on a physical level) affects CY. In humans, there is ample evidence that the more similar that individuals are in terms of physical characteristics, but also personal convictions and views, the more likely they are to automatically mimic behavior^85^.

Future studies can improve on the current study design in several ways. First, we only used orangutan males as stimuli. In previous studies with chimpanzees^10^ and bonobos^15^, the sex of the triggering yawner affected the occurrence of CY; i.e. in chimpanzees, male yawns were more contagious whereas in bonobos, female yawns were more contagious. In gelada baboons, CY is more prevalent among females, especially when they are closely bonded^6^. It is possible that these results can be explained by emotional closeness between individuals, as in chimpanzees males typically form strong social relationships^86^, and in bonobos and gelada baboons it is mostly females that bond^87,88^. Alternatively, results could be explained by the differences in hierarchy with chimpanzees being male dominant^89^ and bonobos female dominant^87^, and by the strong matrilineal bonds between gelada baboons^90^. Investigating whether there is an interaction between sex of the stimulus and of the responder in orangutans could help elucidate the roots of the observed sex effects in CY in some species. The restricted selection of stimuli and the low sample size did unfortunately not allow us to perform such analyses. It is noteworthy, however, that the males in our study yawned more frequently than the females (i.e. the total yawning rate of males was 74, whereas females yawned only 9 times. See Table S1a). Yawns occur more frequently in males of species with canine polymorphism, and also during aggressive contexts^91^. Given that all our stimuli were male, perhaps there is a role for dominance or rivalry in the occurrence of CY in orangutans^23^. Nevertheless, one could argue that this leads to tense situations, thus leading to more scratching when observing yawns of others, which is not what we found.

Additionally, all of our videos contained flanged males. Flanged adult males are often preferred over unflanged males by receptive female orangutans^92^, and can be viewed as threatening by unflanged males^93^. As such, in addition to interactions between the different sexes and CY, it may also be interesting to study potential effects of the two different morphs of orangutan males on CY.

Furthermore, due to power issues, we could not reliably test effects of age on CY. In humans, while spontaneous yawns can occur already before birth^94^, CY does not seem to appear until the age of four to five^95,96^, although when children of 3 years old are specifically told to look at the eyes of the stimulus they show CY as well^97^. Similar developmental trajectories of CY have been reported in other animals^6,7,11,50^. In our study, there were only two individuals younger than 5; one 15 months (Indah) and one three-year old (Baju). We observed one yawn occurrence in Indah (in the yawn condition), in Baju we observed six events (four in the yawn and two in the control condition). We decided to include these individuals in our study because while it is true that CY shows a relatively slow developmental pattern in humans, orangutans are born more precocial, and developmental rates in nonhuman primates are much faster compared to humans^98^. Therefore, CY may possibly also occur earlier in development in orangutans, but with only anecdotal evidence we cannot verify this in our study.

Third, while we tested effects of familiarity in our study by including both familiar and unfamiliar yawners, the fact that we only had yawns from the two adult males to use as stimuli restricted any potential investigation of the potential link between social closeness of the responders and the familiar individuals on the stimuli. The positive effect of social closeness on the occurrence of CY is well established in humans^99^, chimpanzees (but see^10^), and bonobos^15^, but is strongly debated in other species such as dogs^45^ and budgerigars^51^. For dogs, it should be noted that CY is interspecific, and that domestication might have had influential effects on how CY is modulated. Inverse effects have also been reported. For instance, a large study in rats has shown a familiarity bias in the opposite direction with rats being more likely to yawn in response to unfamiliar yawns^23^. Similarly, a recent study investigating scratch contagion in orangutans found that during tense situations, orangutans are more likely to take over scratching from individuals with whom they have a weak bond^80^, indicating a (negative) correlation between social closeness and the contagiousness of a behavior or motor pattern. Thus, it remains possible that social modulation of CY is present in orangutans, at least in those living with conspecifics in captivity, although its presence was not shown in our sample. Yet, given our small sample size, replications that test for the presence and subsequent direction of social modulation of CY in orangutans are needed.

Finally, we could not quantify attention to the screen, which is one of the common methodological issues raised by Massen et al. (2017). We tried to maximize attention to the screen by using attention-grabbing videos of caretakers at the start of every video sequence, and by adding colored screens in-between stimulus presentations. Furthermore, we made sure that orangutans had a direct line of sight towards the screen at the start of the experiment, and only recorded yawns when they directed their attention to the screen at least once during stimulus presentation. Nevertheless, quantification of attention to the stimuli (either measured as a continuous variable or a frequency of gazes) remains the most robust way to control for potential effects of attentional bias.

To summarize, our findings contribute to understanding the evolutionary basis of CY in hominids by showing that orangutans, like humans, chimpanzees and bonobos, yawn contagiously.

## Supplementary Information

Supplementary Information.

## Data Availability

All data, code, and materials that are associated with this paper and used to conduct the analyses are uploaded and made openly accessible on the archiving platform DataverseNL: https://doi.org/10.34894/JIWWCN.
