# Supplementary material for: Experimental evidence for yawn contagion in orangutans (Pongo pygmaeus)
Source: Sci Rep. 2020 Dec 17;10:22251. doi: 10.1038/s41598-020-79160-x (PMC7747555; doi:10.1038/s41598-020-79160-x)
Supplement: Supplementary file 1 — Supplementary Information. [file 41598_2020_79160_MOESM1_ESM.docx]

**Experimental evidence for yawn contagion in orangutans (*Pongo pygmaeus*)**

Evy van Berlo^1,2*^, Alejandra P. Díaz-Loyo^3^, Oscar E. Juárez-Mora^3^, Mariska E. Kret^1,2^, Jorg J. M. Massen^4^

1 Leiden University, Institute of Psychology, Cognitive Psychology Unit, Leiden, The Netherlands
2 Leiden Institute for Brain and Cognition (LIBC), Leiden, The Netherlands
3 Laboratorio de Ecología de la Conducta, Instituto de Fisiología, Benemérita Universidad Autónoma de Puebla, Puebla, Mexico.
4. Department of Biology, Animal Ecology Group, Utrecht University, The Netherlands

* Corresponding author: Evy van Berlo, [e.van.berlo@fsw.leidenuniv.nl](mailto:e.van.berlo@fsw.leidenuniv.nl)

**Supplementary Methods**

**
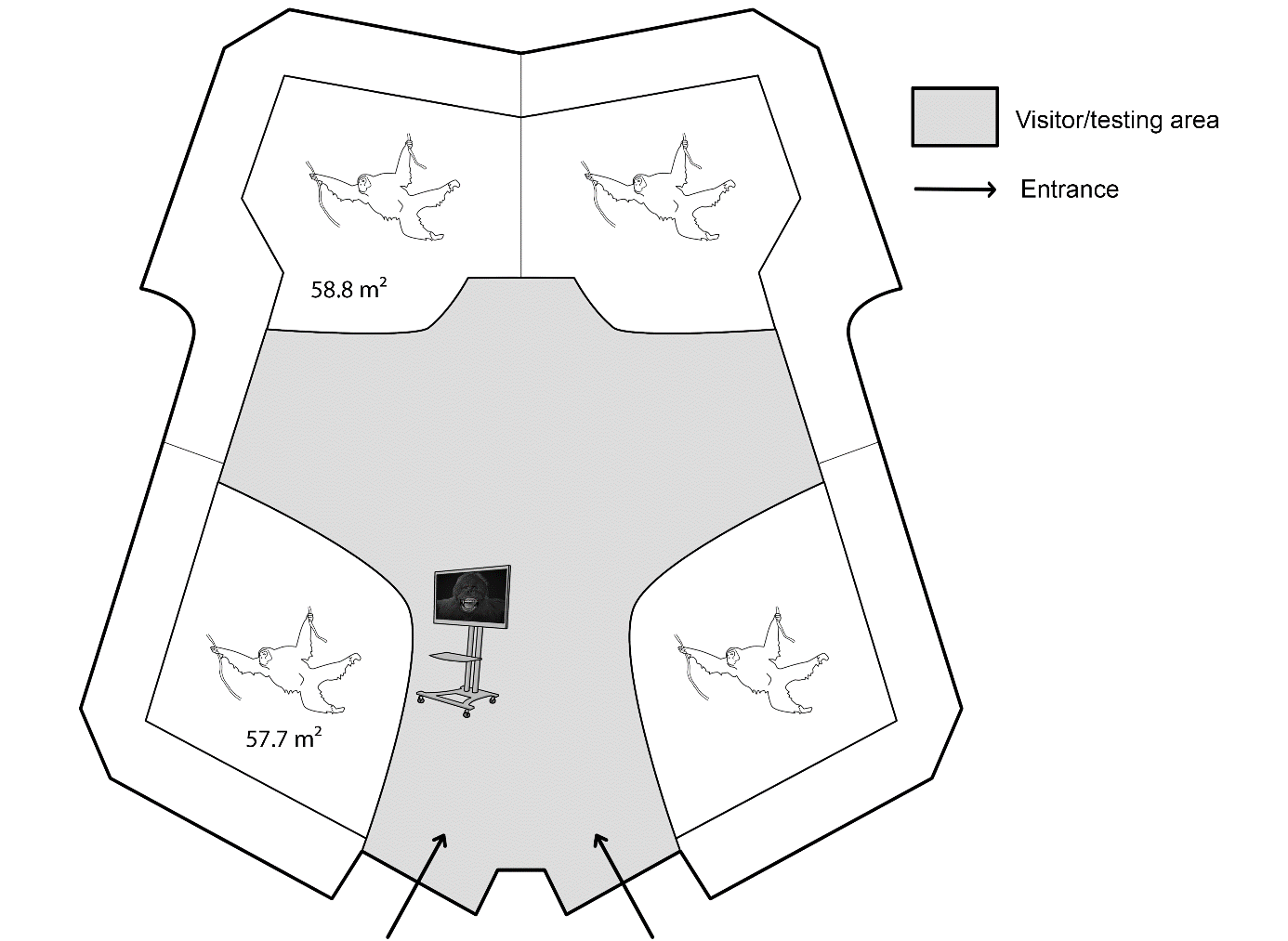
**

**Figure S1.** Abstract representation of the testing area and group composition. Experiments took place in the visitor area but while the park was closed to visitors. The TV was an LG 47LH5000, 47”, 1920x1080 pixels, to which the orangutans were habituated before commencing testing. Only the experimenters and, occasionally, a caretaker, were present. The screen was always directed at one of the four enclosures, which prevented orangutans in the other enclosures from seeing the videos. The two females present in Apenheul were housed with their offspring and sometimes with one adult male. Food was provided four to six times a day and consisted of a variety of vegetables, and sometimes nuts, hay, and fruit, hidden in the enclosure for foraging purposes. Water was available ad libitum.

***Procedure: time, duration, and housing situation***

Test sessions were carried out in mornings and afternoons (between 10:00-15:30) in the inside enclosures (Figure S1). Testing took place between 21-01-2019 and 13-03-2019. At time of testing, the park was closed for visitors. As such, mainly two observers and occasionally a zookeeper were present during testing. Sometimes, volunteer guides belonging to the park visited the orangutans during testing. Furthermore, nearing the end of the testing period one of the individuals had fallen ill and therefore two caretakers were present during testing almost daily. At the beginning of every session, the screen was placed in front of one of the enclosures. An enclosure usually housed one to four individuals (often mother-offspring and one adult male), and testing only started when the focal individual was in a position that allowed it to see the screen. Furthermore, if a focal individual did not see at least one full clip (yawn or control), another attempt was made by moving the screen or waiting until the focal was in a suitable position. All individuals in the enclosure were filmed with two cameras. If the focal individual moved, the observers adjusted the position of the screen as well.
 Yawns that occurred during the primer or while it was clear that the subject had no direct line of sight towards the screen when the stimuli were presented, were recorded as spontaneous yawns and not taken into account in subsequent analyses. Note however, that the number of spontaneous yawns was almost exactly equal between the two conditions (18 and 17 in the yawn and control condition, respectively).

**Supplementary results**

**Table S1a.** Overview of yawning *occurrences* per individual in yawn and control conditions and across triggers.

| **Trigger** | **Familiar** | | **Unfamiliar** | | **Avatar** | |  |
| --- | --- | --- | --- | --- | --- | --- | --- |
| **Individual** | **Yawns in Control** | **Yawns in Yawn** | **Yawns in Control** | **Yawns in Yawn** | **Yawns in Control** | **Yawns in Yawn** | **Total** |
| Amos ♂ | 2 | 6 | 0 | 1 | 2 | 1 | 12 |
| Baju ♂ | 1 | 2 | 0 | 0 | 1 | 2 | 6 |
| Indah ♀ | 0 | 0 | 0 | 1 | 0 | 0 | 1 |
| Kawan ♂ | 2 | 2 | 6 | 6 | 1 | 1 | 18 |
| Kevin ♂ | 0 | 2 | 0 | 1 | 0 | 0 | 3 |
| Samboja ♀ | 0 | 0 | 0 | 0 | 0 | 0 | 0 |
| Sandy ♀ | 0 | 1 | 0 | 1 | 0 | 0 | 2 |
| Wattana ♀ | 0 | 1 | 0 | 1 | 0 | 1 | 3 |
| **Total** | **5** | **14** | **6** | **11** | **4** | **5** | **45** |

**Table S1b.** Overview of yawning *rates* per individual in yawn and control conditions and across triggers.

| **Trigger** | **Familiar** | | **Unfamiliar** | | **Avatar** | |  |
| --- | --- | --- | --- | --- | --- | --- | --- |
| **Individual** | **Yawns in Control** | **Yawns in Yawn** | **Yawns in Control** | **Yawns in Yawn** | **Yawns in Control** | **Yawns in Yawn** | **Total** |
| Amos ♂ | 2 | 8 | 0 | 1 | 2 | 1 | 14 |
| Baju ♂ | 3 | 8 | 0 | 0 | 2 | 4 | 17 |
| Indah ♀ | 0 | 0 | 0 | 1 | 0 | 0 | 1 |
| Kawan ♂ | 2 | 4 | 9 | 21 | 1 | 1 | 38 |
| Kevin ♂ | 0 | 2 | 0 | 3 | 0 | 0 | 5 |
| Samboja ♀ | 0 | 0 | 0 | 0 | 0 | 0 | 0 |
| Sandy ♀ | 0 | 4 | 0 | 1 | 0 | 0 | 5 |
| Wattana ♀ | 0 | 1 | 0 | 1 | 0 | 1 | 3 |
| **Total** | **7** | **27** | **9** | **28** | **5** | **7** | **83** |

**Table S1c.** Overview of overall yawning *occurrences* in the yawn and control condition per replication cycle

| **Replication** | **Control** | **Yawn** | **Total** |
| --- | --- | --- | --- |
| 1 | 7 | 8 | 15 |
| 2 | 1 | 7 | 8 |
| 3 | 2 | 6 | 8 |
| 4 | 5 | 9 | 14 |
| **Total** | **15** | **30** | **45** |

**Table S1d.** Overview of overall yawning *rates* in the yawn and control condition per replication cycle.

| **Replication** | **Control** | **Yawn** | **Total** |
| --- | --- | --- | --- |
| 1 | 7 | 16 | 23 |
| 2 | 1 | 11 | 12 |
| 3 | 3 | 17 | 20 |
| 4 | 10 | 18 | 28 |
| **Total** | **21** | **62** | **83** |

**Table S2.** Overview of number of trials per individual, per trigger and per condition.

| **Trigger** | **Familiar** | | **Unfamiliar** | | **Avatar** | |  |
| --- | --- | --- | --- | --- | --- | --- | --- |
| **Individual** | **Control** | **Yawn** | **Control** | **Yawn** | **Control** | **Yawn** | **Total** |
| Amos ♂ | 17 | 14 | 13 | 11 | 13 | 15 | 83 |
| *Baju ♂ | 9 | 6 | 9 | 6 | 4 | 10 | 44 |
| *Indah ♀ | 14 | 14 | 13 | 16 | 11 | 11 | 79 |
| *Kawan ♂ | 19 | 18 | 21 | 19 | 18 | 18 | 113 |
| Kevin ♂ | 17 | 14 | 17 | 15 | 15 | 16 | 94 |
| Samboja ♀ | 14 | 16 | 14 | 15 | 17 | 15 | 91 |
| Sandy ♀ | 15 | 19 | 16 | 18 | 14 | 13 | 95 |
| Wattana ♀ | 18 | 12 | 19 | 18 | 16 | 18 | 101 |
| **Total** | **123** | **113** | **122** | **118** | **108** | **116** | **700** |

** Baju and Kawan are the offspring of Wattana. Indah is the offspring of Samboja. Note: The numbers are not equally divided across individuals, as bystanders were present in the same enclosure as the focal were also exposed to the videos (for instance in the case of mother-offspring pairs). Furthermore, within individuals, numbers are not equal between conditions because in some cases the focal individual paid no attention to the screen, leading to no data for these particular trials. Finally, one video was accidentally presented an extra time, which means that after data collection we had 289 rather than the planned 288 sessions.*

**Table S3.** Results of Intraclass Correlation Calculation on yawning, absolute-agreement, 2-way random-effects model

|  |  | 95% Confidence Interval | | F Test with True Value 0 | | | |
| --- | --- | --- | --- | --- | --- | --- | --- |
|  | ICC | Lower bound | Upper bound | Value | d1 | df2 | *p* |
| Average measures | .764 | .57 | .87 | 4.23 | 43 | 43 | <.001 |

**Table S4.** Results of Intraclass Correlation Calculation on scratching, absolute-agreement, 2-way random-effects model

|  |  | 95% Confidence Interval | | F Test with True Value 0 | | | |
| --- | --- | --- | --- | --- | --- | --- | --- |
|  | ICC | Lower bound | Upper bound | Value | d1 | df2 | *p* |
| Average measures | .894 | .806 | .942 | 9.40 | 43 | 43 | <.001 |

**Supplementary Analyses**

***Testing the link between yawn occurrence, condition and familiar and unfamiliar trigger (excluding avatar)***

As CY seemed to be present only in response to a familiar or unfamiliar trigger, but not significantly so with the avatar trigger, we performed an extra exploratory analysis without the avatar trigger. Specifically, we first tested whether yawn occurrence is moderated by *condition* (yawn vs. control), *trigger* (familiar vs. unfamiliar), and their interaction using a binomial GLMM with *subject* nested in *trial*. Next, in those cases that at least one yawn occurred, we tested whether yawning rate is moderated by *condition, trigger,* and their interaction using a negative binomial GLMM with *subject* nested in *trial*.
 In the first analysis looking at the likelihood of yawning, we re-confirmed the presence of CY in the reduced dataset: we found a main effect of *condition* (*β* = 6.74, *SE* = 1.65, *Z* = 4.082, *p* < .001) in which individuals were more likely to yawn in the yawn vs. control condition. We did not find evidence for an interaction between *condition*trigger* (*β* = -3.24, *SE* 2.05, *Z* = 1.57, *p* = .115). Despite our reduced dataset, we still confirm the presence of CY in orangutans, but find no evidence for a familiarity effect using ‘real’ orangutan stimuli only.
 In the second analysis in which we looked at yawning rates, we found no significant main effect of *condition* (*β* = .37, *SE* = .46, *Z* = .81, *p* = .420), nor an interaction effect between *condition*trigger (β* = .32, SE = .61, Z = .52, *p* = .603). Similar to the original analysis including the avatar trigger, we find no differences in strength between conditions and triggers. The model including the interaction between *condition* and *trigger* did not significantly improve the null model (*χ2*(3) = 3.50, *p* = .321).

***Testing the link between contagious yawning, condition and familiarity using scratching as covariate***

To control for scratching in our models that investigate the presence and strength of contagious yawning, we perform two Hurdle models, each containing two analyses. First, using a binomial GLMM with *Subject* nested in *Trial* as random factors and *condition* and *scratch occurrence* as fixed factors, we find a main effect of *condition* on the likelihood of yawning (*β* = 3.52, SE = 1.05, Z = 3.35, p = .0008), but no effect of *scratch occurrence* (*β* **=** 1.33, SE = .96, Z = 1.39, p = .163). In those cases that at least one yawn occurred, is the yawn response larger in the yawn condition versus the control condition? To answer this question, we perform a negative binomial GLMM with *Subject* nested in *Trial* as random factors and *condition* and *scratch rate* as fixed factors and compare this model to the null model (without fixed factors). The result shows that the alternative model cannot explain the data better than the null model: χ2 (2) = 3.32, p = .191; there is no main effect of *condition* (*β* = .47, SE = .26, Z = 1.80, p = .071), nor a main effect of *scratch rate* (*β* = .03, SE = .06, Z = .48, p = .634) on yawn rate.
 In the second hurdle model, we look at effects of familiarity on the occurrence and strength of contagious yawning. First, using a binomial GLMM with *Subject* nested in *Trial* as random factors and *condition,* *trigger, condition*trigger,* and *scratch occurrence* as fixed factors, we find a main effect of *condition* and *condition*trigger* on the likelihood of yawning, but no effect of *scratch occurrence* (*β* **=** 1.33, SE = .96, Z = 1.39, p = .163). Specifically, yawning is more likely to occur in the yawn versus control condition in case of a familiar trigger (*β* **=** -6.50, SE = 1.64, Z = -3.97, p = .0001) and an unfamiliar trigger (*β* = -3.93, SE = 1.57, Z = -2.50, p = .012). Next, in those cases that at least one yawn occurred, is the yawn response larger in the yawn condition versus the control condition and does familiarity affect this result? To answer these questions, we perform a negative binomial GLMM with *Subject* nested in *Trial* as random factors and *condition*, *trigger, condition*trigger,* and *scratch rate* as fixed factors and compare this model to its respective null model. The result shows that the alternative model cannot explain the data better than the null model: χ2 (6) = 5.30, p = .505; there is no main effect of *condition* (*β* = .18, SE = .62, Z = .28, p = .776), nor a main effect of *scratch rate* (*β* = .04, SE = .07, Z = .58, p = .560), nor an interaction between *condition*trigger* (*β* = .19, SE = .76, Z = .26, p = .798) on yawn rate.
 As such, we confirm that orangutans yawn contagiously in response to ‘real’ orangutan stimuli, regardless of whether they are familiar or not. Furthermore, we cannot draw any conclusions on the rate of yawning. Importantly, scratching does not have a significant impact on the occurrence of the aforementioned results.
